# Biomechanical performance of the Bicaval Transcatheter System for the treatment of severe tricuspid regurgitation

**DOI:** 10.3389/fbioe.2023.1179774

**Published:** 2023-05-18

**Authors:** Fabrizio Crascì, Stefano Cannata, Giovanni Gentile, Caterina Gandolfo, Salvatore Pasta

**Affiliations:** ^1^ Department of Engineering, Università degli Studi di Palermo, Palermo, Italy; ^2^ Department of Research, IRCCS-ISMETT, Palermo, Italy; ^3^ Department for the Treatment and Study of Cardiothoracic Diseases and Cardiothoracic Transplantation, IRCCS-ISMETT, Palermo, Italy; ^4^ Radiology Unit, Department of Diagnostic and Therapeutic Services, IRCCS-ISMETT, Palermo, Italy

**Keywords:** tricuspid regurgitation, transcatheter valve therapies, bicaval valve implantation, finite-element analysis, smoothed-particle hydrodynamics, computational fluid dynamics

## Abstract

**Introduction:** Tricuspid regurgitation (TR) is a relatively common valvular disease, which can result from structural abnormalities of any anatomic part of the tricuspid valve. Severe TR is linked to congestive heart failure and hemodynamic impairment, resulting in high mortality when repaired by elective surgery. This study was undertaken to quantify the structural and hemodynamic performance of the novel Transcatheter Bicaval Valves System (TricValve) percutaneously implanted in the superior vena cava (SVC) and inferior vena cava (IVC) of two patients with severe TR and venous congestion.

**Methods:** After developing the SVC and IVC device models, the contact pressure exerted on the vena cava wall was obtained by computational analysis. Both smoothed-particle hydrodynamics (SPH) and computational fluid dynamics were carried out to quantify caval reflux in the right atrium and the pressure field of pre- and post-TricValve scenarios, respectively.

**Results:** Analysis of contact pressure highlighted the main anchoring area of the SVC device occurring near the SVC device belly, while the IVC device exerted pronounced forces in the device’s proximal and distal parts. SPH-related flow velocities revealed the absence of caval reflux, and a decrease in time-averaged pressure was observed near the SVC and IVC after TricValve implantation.

**Discussion:** Findings demonstrated the potential of computational tools for enhancing our understanding of the biomechanical performance of structural tricuspid valve interventions and improving the way we design next-generation transcatheter therapies to treat the tricuspid valve with heterotopic caval valve implantation.

## 1 Introduction

Tricuspid regurgitation (TR) is a valvular disease with poor short-to medium-term clinical outcomes and is linked to a dilated tricuspid annulus and right ventricle enlargement and dysfunction (Kadri et al., 2019; Topilsky et al., 2019). These detrimental conditions are also characterized by increased left atrial pressure, pulmonary hypertension, and increased right ventricular afterload (Dahou et al., 2019). No major hemodynamic disturbances occur for trivial and moderate TR, since the increase in blood pressure is compensated by the compliant nature of the right atrium. In the case of severe TR, medical management is usually ineffective and can lead to a 5-year mortality rate of nearly 50% (Fender et al., 2019). Correction of the TR condition is of great importance, but since elective surgery has a high risk of mortality, it is contraindicated in this patient population (Fender et al., 2019). Moreover, the optimal timing of surgical repair or replacement still remains an open challenge (Fender et al., 2018).

Fortunately, transcatheter tricuspid valve therapy has recently emerged as a valid option for treating TR, given the development of bioprostheses with specific design features to accommodate the tricuspid valve anatomy (Romeo et al., 2022). Both repair and replacement strategies are available (Curio et al., 2019). For instance, the TriClip Transcatheter Edge-to-Edge Repair device (Abbott Cardiovascular, Plymouth, MN, United States) and the PASCAL Repair system (Edwards Lifesciences, Irvine, CA, United States.) can be adopted to repair the regurgitant tricuspid valve (Romeo et al., 2022). As for percutaneous replacement strategies, early clinical applications relied on devices that were not specifically designed for the tricuspid valve anatomy, such as the SAPIEN 3, which was applied for the first time in 2011 to treat severe TR (Van Garsse et al., 2011). The first use of *ad hoc* devices for treating the tricuspid valve anatomy was reported in 2020 using the self-expanding EVOQUE tricuspid valve replacement system (Edwards Lifesciences, Irvine, CA, U.S.A.) (Fam et al., 2020).

A feature of transcatheter tricuspid valve therapy is the implantation of devices in the central venous position at the level of the cavoatrial junction, thereby adopting a heterotopic approach (Altisent et al., 2021). The rationale for using caval valve implantation is to alleviate congestive signs of heart failure and the hemodynamic impairment of severe TR with venous congestion. The approach is simple and thus evolved quickly with the development of dedicated devices for implantation in the superior and inferior vena cava (Figulla et al., 2016; Toggweiler et al., 2018). The transcatheter bicaval system (TricValve) is the first of its type to be used to treat TR by reducing caval pressure and improves functional status after 8 weeks from implantation (Lauten et al., 2011; Lauten et al., 2012). The TricValve is a heterotopic device composed of two valves, one implanted in the superior vena cava (SVC) and the second one in the inferior vena cava (IVC). Device placement prevents regurgitant flow in the vena system, reducing liver congestion, increasing right ventricle stroke volume into the pulmonary system and improving cardiac output. The TricValve system, which obtained the CE mark in May 2021, allows further treatment on the tricuspid valve by edge-to-edge repair or valve-in-valve replacement.

This study aims to report patient-specific computational investigations of the hemodynamic and structural performance of the TricValve system delivered in two patients with severe TR and venous congestion. After developing virtual models of both SVC and IVC devices, the deployment was simulated to quantify the contact pressure exerted on the vena caval wall as a potential indicator of device migration. Both smoothed-particle hydrodynamics (SPH) and computational flow analysis were carried out to quantify the likelihood of caval reflux near the TricValve system and the pressure field of pre- and post-TricValve implantation in the right atrium, respectively.

## 2 Materials and methods

### 2.1 Patient reconstruction

Two patients who underwent bicaval transcatheter treatment with the TricValve system (*p* + F Products + Features, Germany) were investigated. The case of a 72-year-old male patient with severe TR and signs of heart failure was investigated (Case A). The patient had a past history of both aortic and mitral valve repair, as well as pacemaker implantation in the right heart side. Similarly, the second patient was 66 years old with a history of pacemaker implantation and past surgery for both aortic and mitral valve repair (Case B). For both patients, TR and caval reflux with preserved left ventricular function were present. Conventional surgery was highly contraindicated because of advanced age and congestive heart failure conditions.

Pre-procedural computed tomography (CT) was used to reconstruct the right atrium and main branches, including the superior vena cava (SVC) with the right innominate vein, the inferior vena cava (IVC) and the hepatic vein. Anatomy was segmented using semi-automatic thresholding followed by manual editing and smoothing using the medical imaging software Materialise Mimics (v21.0; Materialise, Leuven, Belgium). Patient geometries were then exported to generate the structural model for simulating TricValve deployment as well as the fluid model for evaluating pre- and post-TricValve hemodynamics. Using Ansys ICEM (v2021 Ansys Inc., Canonsburg, PA, U.S.A.), the structural and fluid models were meshed with unstructured triangular elements (size 0.6 mm) and tetrahedral elements (size 0.8 mm), respectively.

### 2.2 Bioprosthesis model

**FIGURE 1:**
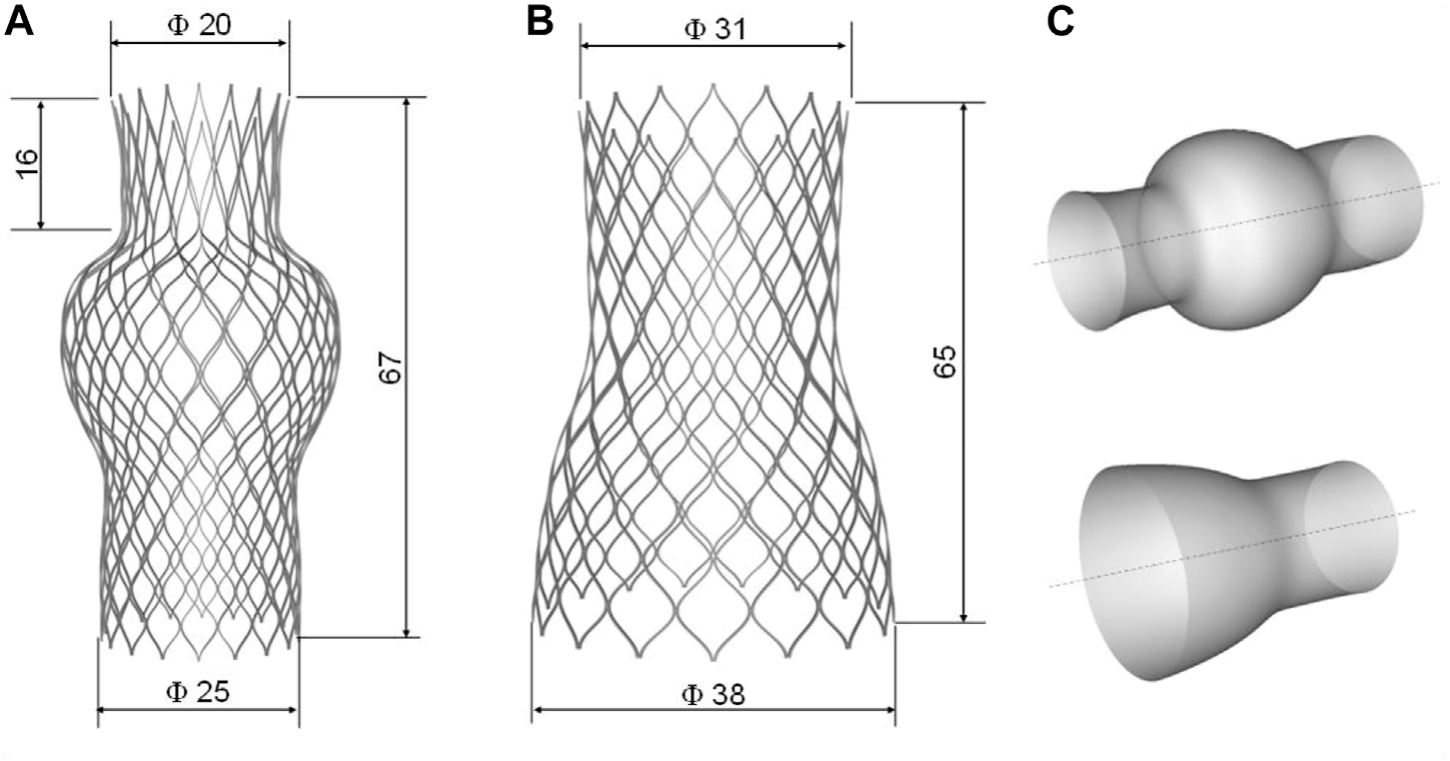
Geometry with main dimension of **(A)** SVC device and **(B)** IVC device as well as the **(C)** primitive surface of each device.

The TricValve system is composed of two self-expanding transcatheter bicaval valves with a nitinol stent frame and valve leaflets made of bovine pericardium sutured onto the stent structure (Figure 1A). Devices are implanted percutaneously in the SVC and IVC walls, and therefore do not alter the native tricuspid valve anatomy. Each device is designed with a sealing skirt of PET material to mitigate the risk of caval reflux. Though the SVC device has a longer skirt than the IVC device, high implantation of the SVC bioprosthesis with the belly part at the level of the innominate plane is recommended to prevent obstruction of the left innominate vein. The deployment of SVC and IVC devices leads to an increase in the cardiac output by reducing the backward regurgitant flow, and thus improves the patient’s functional status at the 8-week follow-up (Lauten et al., 2011).

Device geometries were obtained using photographic images and design data from the manufacturer’s manual. The net of the stent frame was generated starting from the primitive geometry of each device using Rhinoceros (v.7, McNeel & Associates, Seattle, WA, United States). Several control points representing the curvature of the primitive wire frame were interpolated by a degree 3 NURBS curve and mirrored and replicated in polar series. For each device, the unfolded geometry was wrapped around the device surface to obtain the final device shapes (Figure 1C). The IVC device was composed of six cells in the longitudinal direction and sixteen cells in the circumferential direction. The SVC device was modeled with seven cells in the longitudinal direction and eighteen cells in the circumferential direction. The metallic stent frame had a rectangular cross-section of 0.300 × 0.135 mm for both the SVC and IVC devices. The devices were then positioned in the patient’s SVC and IVC according to the manufacturer’s instructions for use and Heart Team indications.

The TricValve nitinol model was meshed with an element size of 0.5 mm resulting in 149,939 and 167,729 hexahedral structured elements, respectively (C3D8). The constitutive model proposed by Auricchio et al. (Auricchio et al., 2014) was adopted to account for the material properties of the TricValve nitinol stent frame. A Rayleigh damping of 200 was adopted to regulate the dynamic material response and a density of 6,590 kg/m^3^ was used. Both the skirt and device valve leaflets were mapped onto the luminal side of the stent frame after device deployment, and then the element overclosures among the different parts were resolved by interference fits in the Abaqus/Explicit solver (v.2021hf7, Dassault Systèmes, FR). A hyperelastic, incompressible material formulation represented by the Ogden material model with *μ* = 0.159 MPa and *α* = 10.89 was adopted to model the pericardial tissue of device valve leaflets, as done by Bailey 2016 (Bailey et al., 2016). The device valve leaflets were assumed to be 0.4 mm thick. The PET material of the device skirt was modeled differently using an elastic-plastic material model and a uniform thickness of 0.1 mm.

### 2.3 Patient-specific models

The deployment of the TricValve system was developed in the Abaqus/Explicit solver platform using a quasi-static approach by limiting element mass scaling to 1.0e-6. This allowed the maintenance of the ratio of kinetic energy to internal energy <10% during all simulation steps. A general contact algorithm was adopted to account for the interaction of the bioprosthesis with the caval wall. Specifically, a penalty formulation with a factor of 0.1 were defined among the parts in contact during the deployment phase (0.5 s).

After device-positioning in the human host, both SVC and IVC devices were crimped using a cylindrical surface and were gradually moved along the radial direction, as done previously in a similar study (Pasta et al., 2020a). Crimpers were meshed with quadrilateral surface elements and had a density of 1,060 kg/m^3^. By pulling the rigid sleeve of each TricValve device towards the right atrium and releasing the device frame, due to the stress field resulting from crimping, the SVC and IVC stent frames were gradually delivered inside the superior and inferior vena cava walls. For boundary conditions, the distal ends of the major vessels of the right atrium were constrained in the longitudinal direction while allowing radial and circumferential displacement. An elastic recoil of 0.1 s ended the delivery of the TricValve system. Given the lack of material properties of the vena cava, we used an incompressible Neo-Hookean model with C1 = 0.6 MPa and D = 0.074 MPa^-1^ and a uniform thickness of 1.5 mm to model the biomechanical response of each patient’s anatomic model (Cosentino et al., 2019; Di Giuseppe et al., 2019). For the sake of simplicity, the atrium was modeled with the same material behavior of the vena cava.

### 2.4 Fluid-solid interaction analysis

**FIGURE 2:**
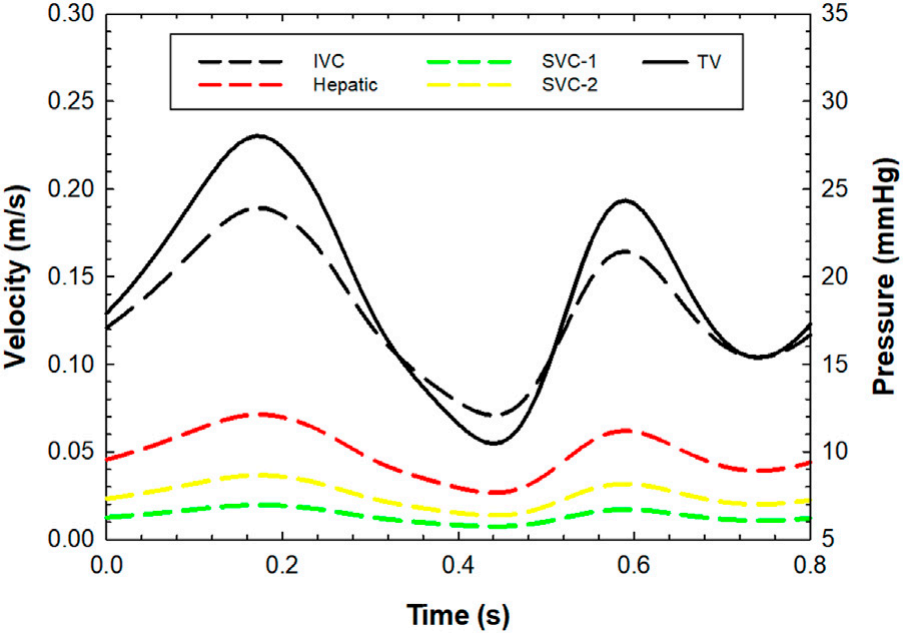
Pre-TricValve flow velocities at inlet for each venous branch and pressure outlet boundary conditions for the tricuspid valve; flow and pressure values were set to zero during the systolic phase (from 0.25 s to 0.55 s) for the post-TricValve flow analysis.

A smoothed-particle hydrodynamics (SPH) analysis was carried out to characterize the fluid-dynamic performance of the TricValve system. This was performed to assess the capability of SVC and IVC devices to seal the right atrium and thus reduce the amount of caval reflux. The SPH analysis was therefore implemented only for Case A. The SPH method is a meshless numerical approach using a general contact formulation between fluid and solid parts and is, therefore, ideal for simulating the opening and closing of the TricValve system (Pasta et al., 2020b). For the fluid, a Newtonian rheological behavior with a density of 1060 kg/m^3^ and viscosity of 0.0035 Pa was modeled using the pressure-density relation governed by the linear Hugoniot equation of state (artificial sound speed of c0 = 145 m/s). For particle discretization, a spatial particle resolution of 0.5 mm was implemented on the basis of a sensitivity analysis performed by Mao and collaborators (Mao et al., 2016). Flow motion was developed by pressure boundary conditions applied on several plate pistons located at the extensions of each branch, using an approach similar to our previous study (Pasta et al., 2020b). The distal ends of the inlet and outlets were extended sufficiently, and then rigid plate pistons were generated for each branch in the normal vessel direction. These pistons were used to apply pressure and motion on either side of the fluid particle because the finite volume of fluid is incompressible. Thus, the physiological pressure waveforms shown in Figure 2 were applied to each piston for generating the blood particle motion using contact conditions and constraining plate rotations. The cardiac beat was 0.8 s long. To develop the FSI analysis, contacts were enabled between the particles and implanted devices, but the caval wall was considered to be rigid. For post-processing, particle flow data were mapped onto a new tetrahedral element mesh of the fluid domain using the Ensight visualization software (v2021 Ansys Inc., Canonsburg, PA, USA) to analyze flow velocity on a contour map instead of discrete particle point collection.

### 2.5 Computational flow analysis

Pre- and post-TricValve hemodynamics were investigated by computational fluid dynamics to quantify the right atrial flow velocity and pressure field of the whole patient model. Using Fluent solver (v2021 Ansys Inc., Canonsburg, PA, United States), laminar flow conditions and non-Newtonian viscosity modeled with the Carreau equation were implemented for the fluid, as done previously (Scardulla et al., 2017). An implicit algorithm with the SIMPLE option for pressure correction and a 2nd-order accuracy upwind scheme was developed to solve the unsteady flow. For boundary conditions, we imposed a flow velocity profile at two SVC branches, the IVC and the hepatic vein using literature data (Figure 2) (de Oliveira et al., 2021). Specifically, the total inflow of 6 L/min was split among inlets with divisions proportional to each inlet area. For the outlet, the pressure waveform was obtained by transforming and scaling the velocity profile to match the cath lab blood pressure measurement of 27 mmHg recorded during the transcatheter bicaval implantation. This pressure profile curve was then imposed at the tricuspid valve as an outlet condition (Figure 2). For the post-TricValve scenario, boundary conditions were estimated considering that both the SVC and IVC device valve leaflets are closed during ventricular systole (i.e., closed shape of tricuspid valve). Thus, the flow velocity was set to null values for the systolic phase (0.3 s) of the cardiac cycle to mimic the absence of caval reflux after device implantation. Three cardiac beats were simulated, and the last cycles were used for flow analysis.

## 3 Results

**FIGURE 3:**
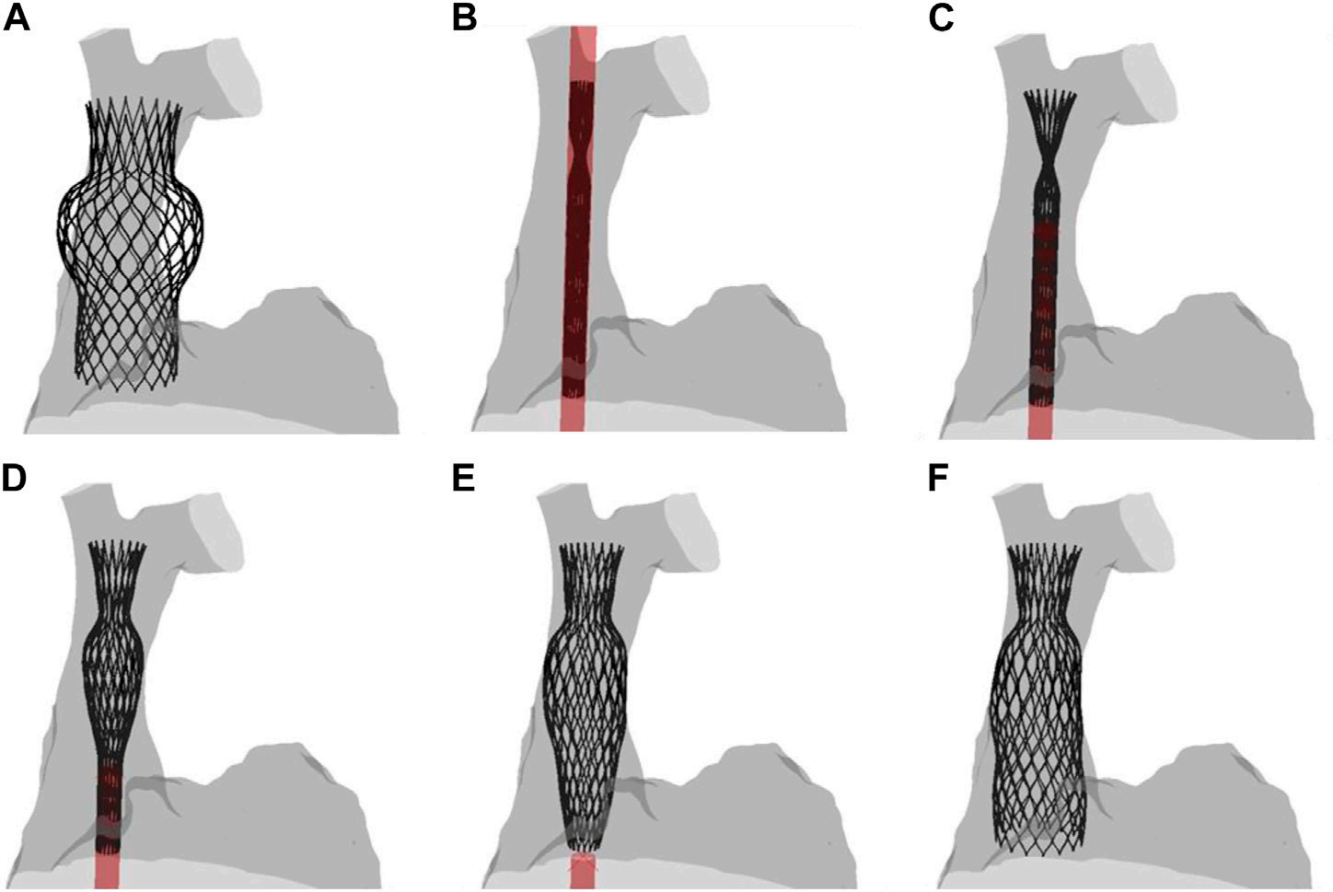
Different phases of the deployment of the SVC device from the **(A)** uncrimped configuration to the **(F)** final deformed shape; intermediate steps of the **(B–E)** deployment are shown.

**FIGURE 4:**
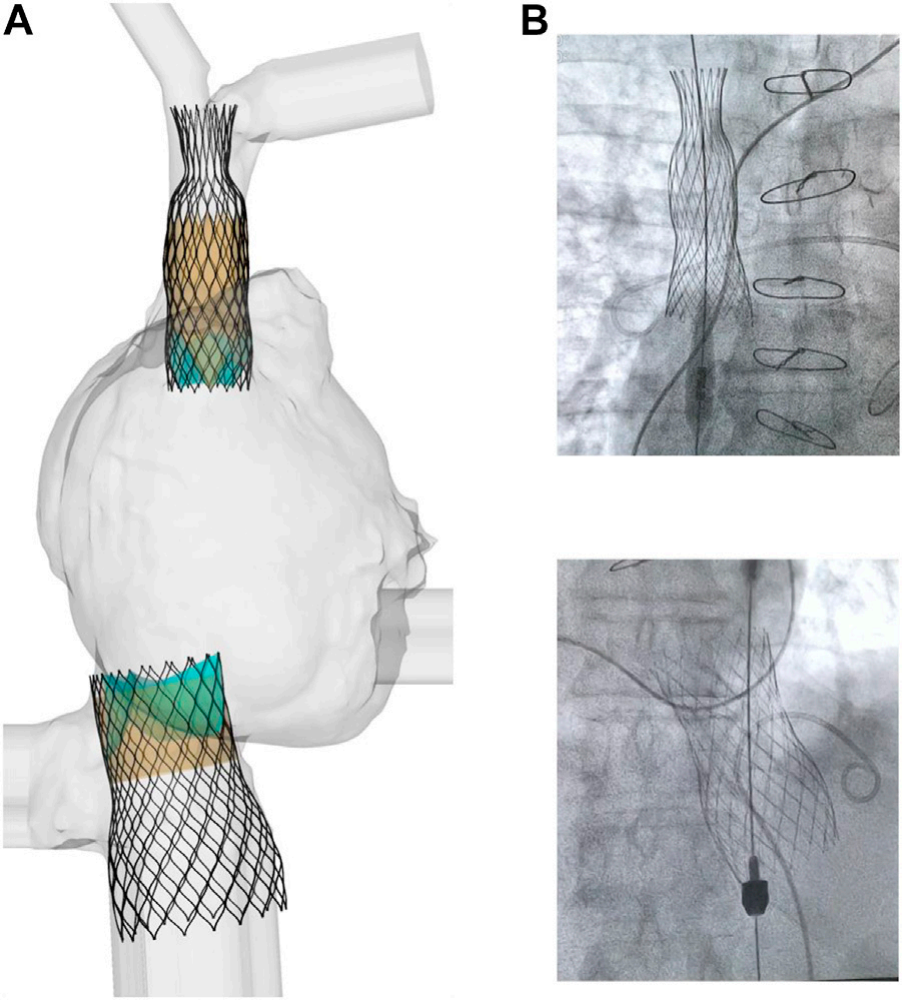
**(A)** deformed shape of implanted TricValve system for Case A and **(B)** angiographic images seen during the clinical procedure for the same patient.

**FIGURE 5:**
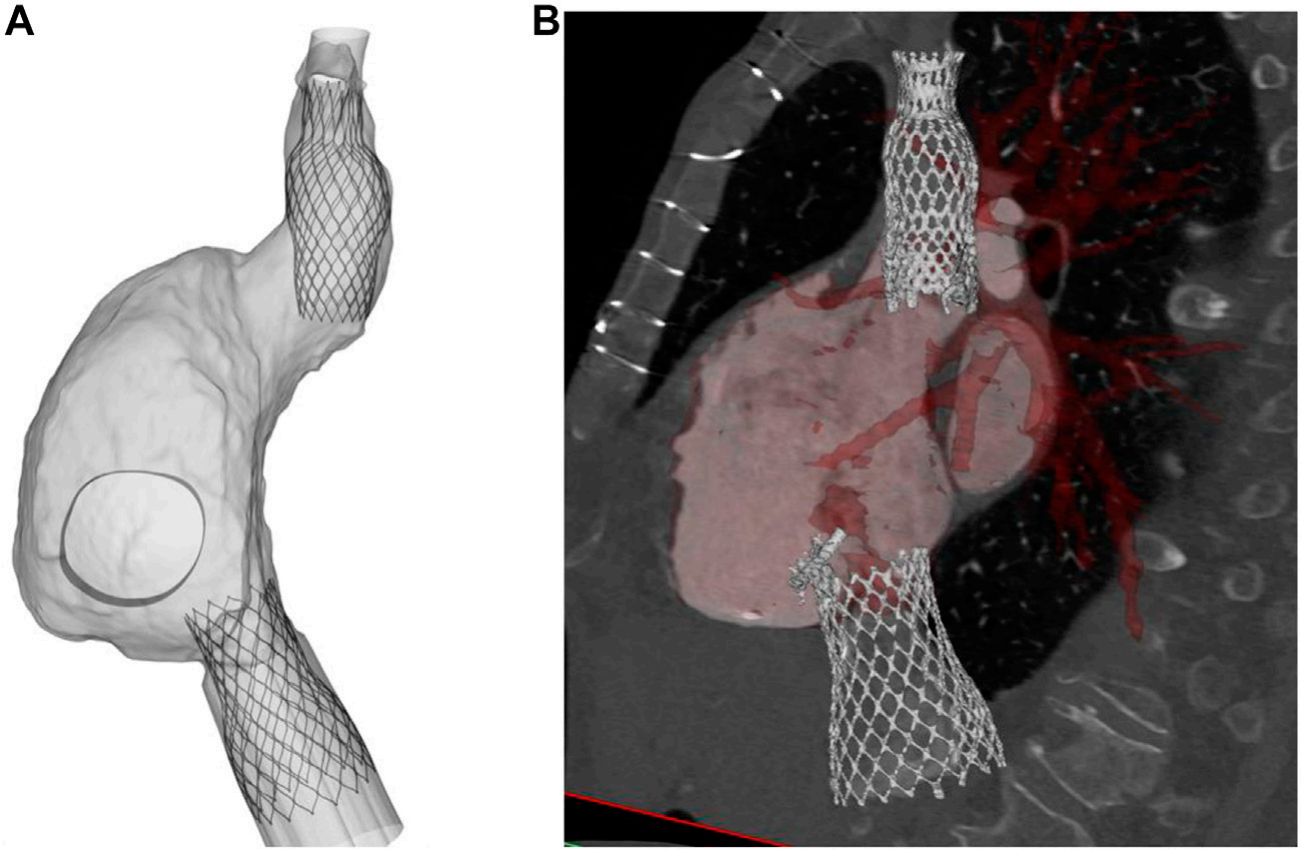
**(A)** deformed shape of implanted TricValve system for Case B and **(B)** CT scan after TricValve implantation.

Figure 3 displays several steps in the IVC device deployment in the patient anatomy of Case A. Once the device was crimped (A), the sleeve was gradually displaced to release the crimped device, exhibiting the main anchoring areas at the mid-level of the venous wall. A qualitative comparison of the computationally-implanted TricValve system with respect to angiographic images was carried out for Case A (Figure 4). The portion of the IVC device facing the hepatic vein was positioned with the proximal device end protruding into the right atrium. Specifically, the device protrusion had a length of 9.75 mm from post-TricValve CT imaging, while the computational simulation reported a device extension of 10.8 mm. Unlike the IVC device, the SVC device illustrated a high implantation depth with the distal end located near the bifurcation of the common jugular vein and the valve leaflets inside the right atrial chamber. A similar implantation configuration was shown by the simulation of Case B, shown in Figure 5. A comparison with the post-TricValve CT imaging was performed for this patient.

**FIGURE 6:**
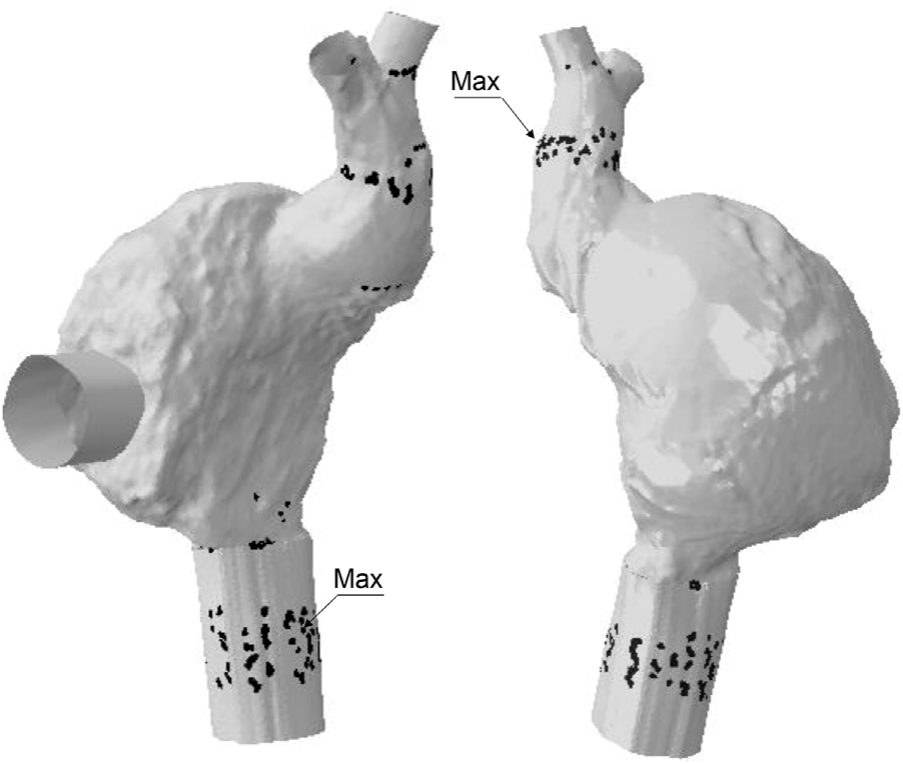
Regions of contact pressure for Case B model; black spots represent area of contact pressure at the magnitude of 0.01 MPa while labels indicates the location of peak values.

The anchoring performance of both SVC and IVC devices was well-characterized by the contact pressure exerted by the device frame on the anatomic wall (Figure 6). For the SVC device, regions of maxima of the contact pressure parameter were observed at the mid-level of the SVC and near the right atrium. However, the IVC device presented two regions of contact pressure peaks corresponding to a) the junction of the inferior vena cava with the right atrium and b) after the bifurcation of the vena cava with the hepatic vein.

**FIGURE 7:**
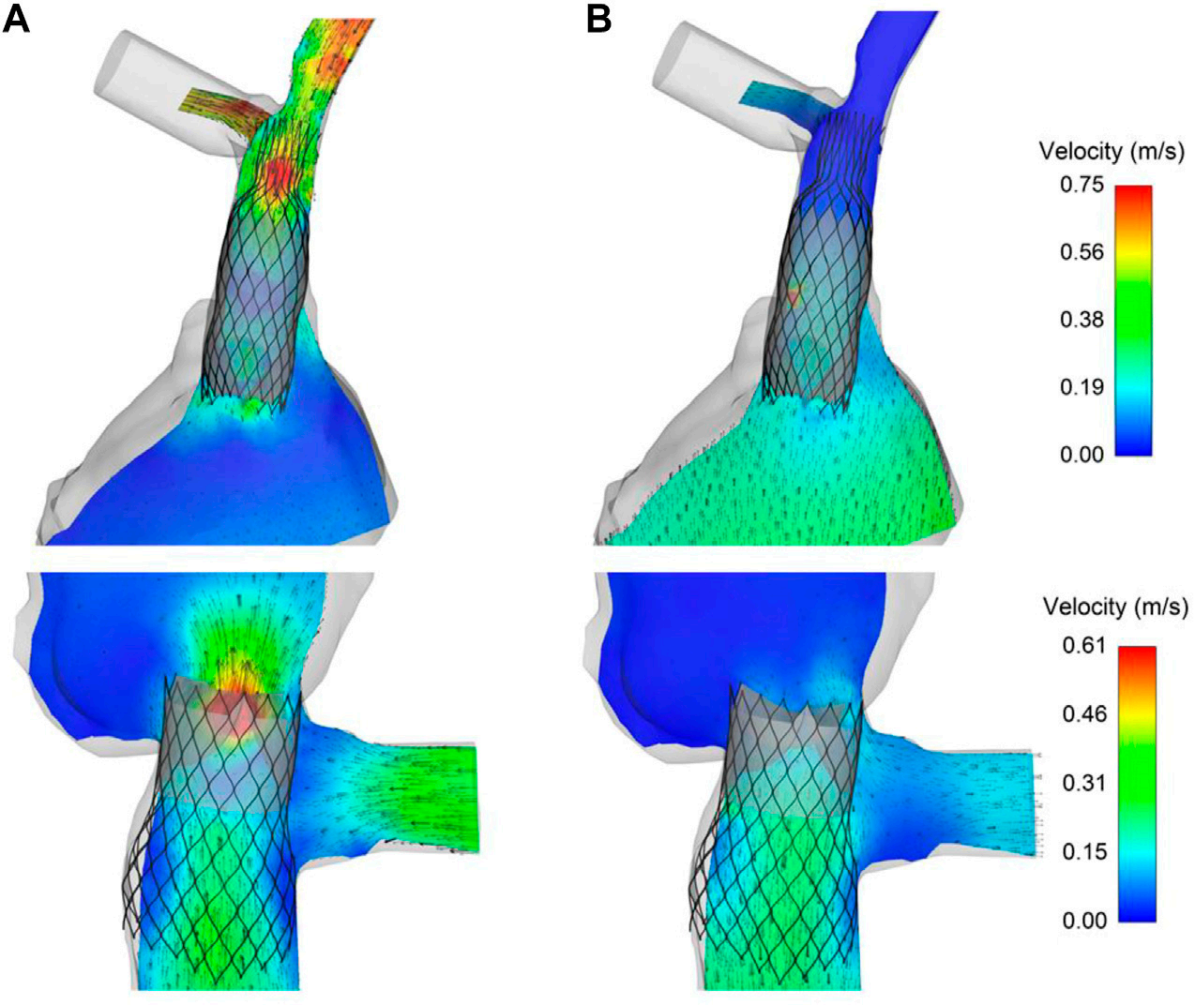
Maps of flow velocities for the TricValve implanted in Case A at **(A)** opened and **(B)** closed configuration of SVC and IVC valve leaflets obtained from SPH analysis.

Maps of flow velocities were computed in cross-sections of both the SVC and IVC devices after SPH analysis to assess the opening and closing of the TricValve leaflets (Figure 7). For the SVC device in Case A, the flow analysis at the opened valve leaflet shape (i.e., ventricular diastole) demonstrated high flow velocity before the bifurcation of the inferior vena cava. Caval reflux appeared limited as low flow velocity occurred in the distal SVC device portion when the valve leaflets were closed. For the IVC device, peaks of flow velocity were found during valve opening, while a paravalvular flow was observed allowing blood circulation in the hepatic vein when the valve leaflets were closed. This was likely caused by the relative position of one of the IVC device valve leaflets facing the large hepatic vein.

**FIGURE 8:**
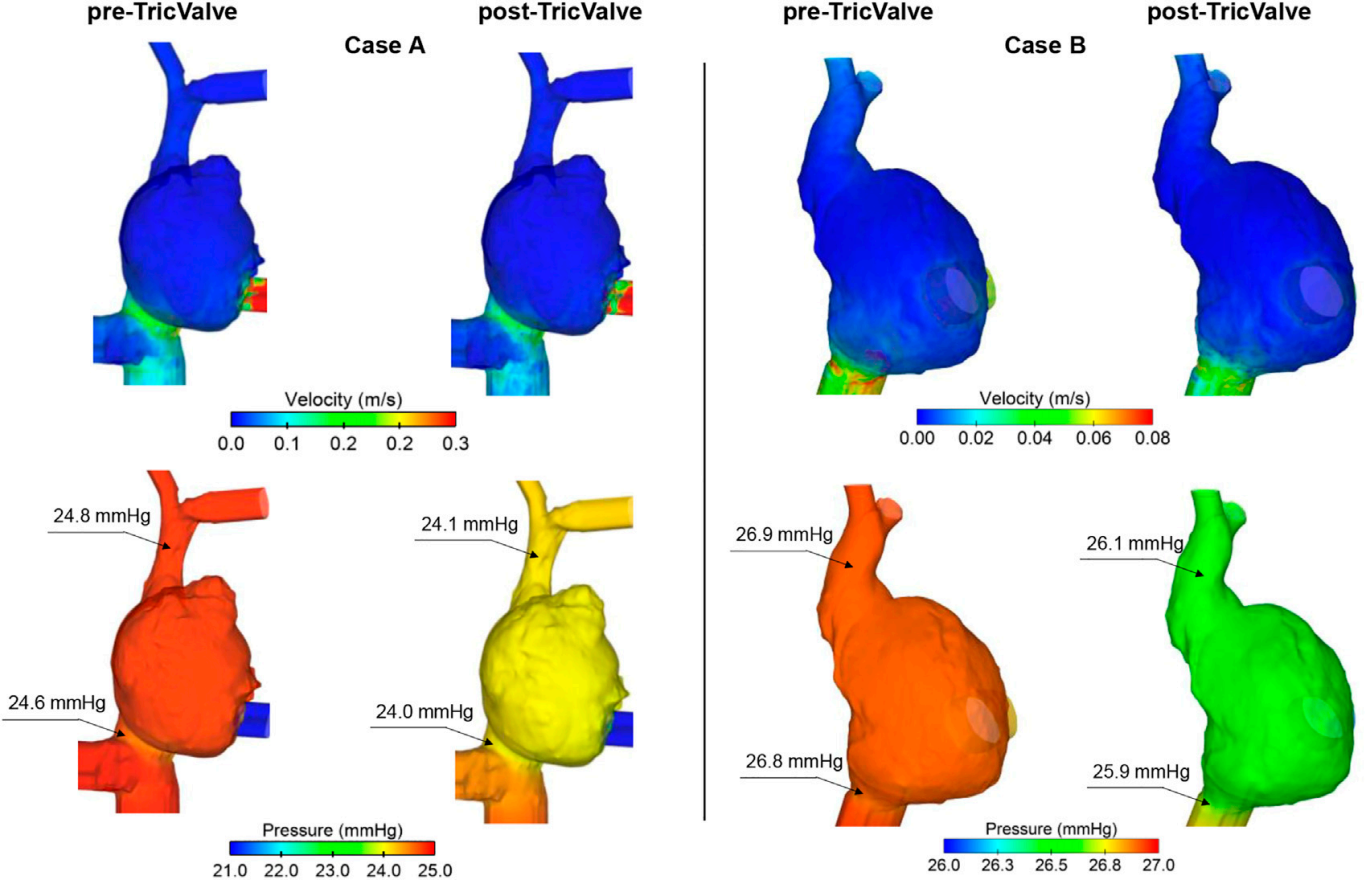
Maps of time-averaged flow and pressure for pre- and post-TricValve computational fluid dynamic analyses of both patient cases.

Figure 8 illustrates the contour map of time-averaged flow and pressure for both pre- and post-TricValve scenarios as obtained from computational fluid dynamics. Time-averaged flow velocity of the pre-TricValve model was high in regions at the ostia of the superior and inferior vena cava, suggesting the likelihood of caval reflux. Flow velocities after device delivery were found to be lower than those occurring prior to device delivery. For Case A, time-averaged pressure on the right atrial wall after device implantation slightly decreased at the mid-level of the right atrium with respect to that seen prior to device implantation (25.1 mmHg for pre-TricValve and 24.5 mmHg for post-TricValve). Similarly, the region of the superior and inferior vena cava showed a slight reduction in blood pressure after device implantation (i.e. 24.8 mmHg for pre-TricValve, and 24.1 mmHg for post-TricValve of SVC for Case A, see inset of Figure 8). Similar flow and pressure fields were observed for Case B.

## 4 Discussion

In this study, computational methodologies permitted us to quantify the hemodynamics and structural performance of the novel TricValve system to treat signs of TR and venous congestion by delivering two *ad hoc* devices designed for heterotopic caval valve implantation. Contact pressure highlighted the anchoring regions of the devices to the venous system, while flow analysis demonstrated a decrease in the pressure field near the SVC and IVC after TricValve implantation and the absence of caval reflux. To our knowledge, these computational findings have never been reported, as clinical evidence has only documented early experience with this novel bicaval device system. The main objective of the TricValve system is to protect the venous system from the elevated right atrial pressure and reduce the systolic caval backflow induced by severe TR, without any correction of the tricuspid valve disease itself (Lauten et al., 2011). This study improves our knowledge of the biomechanics of the TricValve system with the aim of yielding better pre-procedural planning than decisions based only on imaging, and provides insights on how to improve the design of novel transcatheter heart valves by minimizing the reported complications.

Since the first-in-man study of the TricValve system in 2011 (Lauten et al., 2011), several clinical investigations have reported the feasibility of caval valve implantation in treating severe TR for patients deemed to be at extremely high surgical risk (Jin et al., 2022; Kultursay et al., 2022). The procedure was reported to be hemodynamically stable, being characterized by a slow-controlled device delivery and implant time comparable to that of self-expanding stents for transcatheter aortic valve implantation (TAVI) (Altisent et al., 2021). In a multicenter prospective trial in Europe, the safety and efficacy at 6 months from TricValve implantation was demonstrated by favoring right cardiac function (from NYHA functional class III or IV to class I or II) as well as having low mortality and hospitalization rates (in the range of 8.5% and 20%, respectively) (Estevez-Loureiro et al., 2022). As with other self-expanding devices, TricValve system deployment is represented by a complex clinical decision-making process involving two devices with different sizes and specifications on the landing zone and different anatomic constraints. Clinical evidence has demonstrated that the interaction of the device with the caval wall can lead to several risks, such as device migration and leakage (Kultursay et al., 2022). For instance, Kultursay et al. observed the migration of the IVC device into the right atrium just after deployment. They also observed migration of the SVC device, which was managed by delivering another SVC device into the original TricValve system. Imaging remains crucial for understanding the proper device size to be implanted, as the TricValve system tolerates a landing zone ≤35 mm and a suboptimal device size might result in device embolization. In this study, we assessed structural indicators usually seen in computational studies of transcatheter aortic and mitral valve implantation to quantify the biomechanical anchoring performance of the TricValve system (Finotello et al., 2017; Pasta et al., 2020a; Pasta et al., 2020b; Nappi et al., 2021; Pasta et al., 2022). The lower the device migration and leakage is, the higher the wall rupture. In TAVI, Wang et al. (Wang et al., 2015) demonstrated that simulated aortic rupture locations were in agreement with clinical observations or decisions not to operate. Similarly, the distribution of contact pressure between the device and the aortic wall was proposed as an indicator of device anchoring (Morganti et al., 2014; Luraghi et al., 2019). In this study, the SVC-related device forces were pronounced in regions at the mid-level of the SVC wall, thus corresponding with the device belly. This part of SVC device is generated to reduce the risk of obstruction of flow in the innominate vein, with the device skirt usually positioned below the ostia of the innominate vein. This configuration may lead to device migration into the right atrium if contact pressure on the SVC wall is not sufficient enough to withstand hemodynamic forces. Anchoring can also be influenced by the presence of a pacemaker lead in the SVC, which is commonly implanted in this patient population to treat right atrial failure. On the other hand, the IVC device exhibited a peak contact pressure in the distal part as a consequence of the large device diameter resulting in high radial forces on the venous wall. It is known that an exacerbated overexpansion of the IVC device can compress branches of the phrenic nerve and thus cause phrenic pain. Moreover, the portion of the IVC device protruding into the right atrium appeared similar to the bird-beak configuration seen in thoracic endografts associated with complications like endoleaks (Pasta et al., 2016).

From a fluid-dynamic perspective, there are two main differences characterizing the deployment of the TricValve system compared to other self-expanding devices. First, the TricValve system experiences a low pressure field like that of the venous system compared to the physiological pressure values of the arterial circulation. Secondly, the landing zone should not present calcifications or valve leaflets that may alter the adaptation of the self-expanding stent frame on the vessel wall. The concept behind the positioning of the SVC and IVC devices in the vena cava is to diminish the backflow due to the regurgitant tricuspid valve and, thus, the hemodynamic impairment seen in the right atrial chamber. This leads to an increase in cardiac output and portends reverse remodeling of the right atrium usually 8 weeks after device implantation. Therefore, the beneficial effects of device deployment cannot be seen in our computational study. Notwithstanding, the increase in the right atrial pressure after TricValve deployment predicted here is in agreement with catheterization measurements performed just after device delivery in cath labs (Lauten et al., 2011; Altisent et al., 2021). As the blood flow comes back from the tricuspid valve, the device valve leaflets close to prevent backflow into the venous system. This can likely result in an increase in the right atrial pressure and improve patient cardiac function in the follow-up period by positive right heart remodeling. Additionally, we observed a decrease in the pressure field near the SVC and IVC, suggesting a reduction in the effect of the regurgitant flow waveform volume on the venous system. However, this speculation should be confirmed by more complex computational techniques, such as fluid-solid interactions and realistic boundary flow conditions. In this context, Olivera et al. (de Oliveira et al., 2021) have emphasized the lack of computational flow studies on the right atrium with respect to analyses of the left heart hemodynamics. Similarly, Parker et al. (Parker et al., 2022) indicated the need for an accurate turbulence model to account for the flow mixture originating from both the SVC and IVC in the right atrium.

This study is based on several assumptions due to technical challenges and the lack of available information. Specifically, the material properties of patient models were extrapolated from *ex-vivo* tensile testing data of the aorta as human material characterization is not present in the literature. Similarly, the right atrium was modeled using the hyperplastic material law assumed for the vena cava and assuming uniform thickness, thus not considering the heterogenous, active and passive anisotropic behavior of the right heart. Not any intra-atrial blood pressure was applied during TricValve simulation deployment. In the post-TricValve computational flow analysis, the motion of both SVC and IVC device leaflets was not considered and boundary flow conditions were derived from physiological considerations on the deployment of the TricValve system. Combining computational flow analysis with 1D lumped parameter modeling of both the right and left circulation shows great promise in evaluating the impact of the implanted devices on heart function. Specifically, in the case of the TricValve system which restores hemodynamic balance between the right and left circulation, integration with a lumped parameter model can provide valuable insights into the hemodynamic forces exerted on the regurgitant tricuspid valve. The SPH solver is a particle-based method that does not resolve the conventional Navier-Stokes equation governing fluid motion within a solid object. In this study, the SPH technique was chosen because it can be implemented easily within the same solver used for structural deployment, without the need for simultaneous coupling of the fluid physics with the structural part. Additionally, advanced contact conditions were employed to account for the exchange between fluid forces and resulting displacements. It is worth noting that while the SPH approach is useful for modeling FSI, it may not be the best method for quantifying fluid shear stress. However, this was not the objective of the present investigation. FSI analyses based on the Lattice Boltzmann technique will be investigated to better represent the abrupt change in the right atrial hemodynamics following device implantation.

## 5 Conclusion

This study can bring novel insights to the biomechanics of the TricValve system to better understand device anchorage and flow hemodynamics. Findings may also be used to improve the design of novel transcatheter tricuspid valve therapies for the treatment of severe TR in the context of venous congestion.

## Data Availability

The raw data supporting the conclusion of this article will be made available by the authors, without undue reservation.
